# Transdermal Delivery of a Hydrogen Sulphide Donor, ADT-OH Using Aqueous Gel Formulations for the Treatment of Impaired Vascular Function: an *Ex Vivo* Study

**DOI:** 10.1007/s11095-021-03164-z

**Published:** 2022-01-27

**Authors:** Mandeep Kaur Marwah, Hala Shokr, Lissette Sanchez-Aranguren, Raj Kumar Singh Badhan, Keqing Wang, Shakil Ahmad

**Affiliations:** 1 Aston Medical School, College of Health and Life Sciences, Aston University, Birmingham, UK; 2 Pharmacy Division, School of Health Sciences, Manchester University, Manchester, UK; 3 School of Pharmacy, College of Health and Life Sciences, Aston University, Birmingham, UK

**Keywords:** controlled release, hydrogen sulphide donors, release kinetics, skin penetration enhancer, transdermal drug delivery

## Abstract

**Purpose:**

Hydrogen sulphide (H_2_S) is an important signalling molecule involved in the regulation of several physiological and pathophysiological processes. The objective of this study was to investigate the feasibility of transdermal delivery of ADT-OH, a H_2_S donor, by investigating the transdermal flux of aqueous gels loaded with penetration enhancers or liposomes. Furthermore, we explored the ability of permeated ADT-OH to promote angiogenesis and mitochondrial bioenergetics in HUVEC cells.

**Methods:**

Aqueous hypromellose gels (5% w/v) were prepared with up to 10% v/v propylene glycol (PG) or deformable liposomes with 0.025% w/w ADT-OH. ADT-OH permeation from formulations across excised murine skin into PBS was quantified over 24 h using HPLC-UV detection. Media was collected and applied to HUVEC cells to evidence ADT-OH functionality following permeation. Tube formation assays were performed as indicative of angiogenesis and mitochondrial oxygen consumption was evaluated using a Seahorse XF24.

**Results:**

Increasing the loading of PG caused an increase in ADT-OH permeation rate across skin and a decrease in dermal drug retention whereas liposomal gels produced a slow-release profile. Treatment of HUVEC’s using conditioned media collected from the ADT-OH loaded permeation studies enhanced tube formation and the basal oxygen consumption rates after 30 min of treatment.

**Conclusions:**

These findings demonstrate that transdermal delivery of ADT-OH may provide a promising approach in the treatment of impaired vascular function. Gels prepared with 10% v/v PG have the potential for use in conditions requiring rapid H_2_S release whereas liposomal loaded gels for treatment requiring sustained H_2_S release.

**Supplementary Information:**

The online version contains supplementary material available at 10.1007/s11095-021-03164-z.

## Introduction

Hydrogen sulphide (H_2_S) is a central signalling molecule involved in the regulation of several physiological and pathophysiological processes, including cognitive pathways, inflammation, reproduction, and the regulation of blood pressure (1–3). H_2_S has also been observed to offer cardio protection in an irreversible ischemia/reperfusion injury animal model whilst exerting a vasorelaxant effect in various vascular tissues (4). Its valuable effect in the cardiovascular system is comparable to that of nitric oxide (NO) without the harmful production of reactive oxygen species (ROS) observed with NO (5). Furthermore, H_2_S acts as a scavenger of ROS (6) suggesting that its cardiovascular-protective actions may be superior to those displayed by NO donors. H_2_S donors are being investigated as potential therapeutic options where a lack of its endogenous production has resulted in abnormal clinical presentations (7–9).

H_2_S donors offer potential therapeutic alternatives in multiple conditions including myocardial infarction and heart failure (7, 10). ADT-OH is a promising H_2_S donor shown to inhibit apoptosis and scavenge ROS through the stimulation of glutathione (GSH) and gluthathione-S-transferase(GST)(11). However, there are many practical delivery challenges presented by such donors due to their fast rates of H_2_S release, the gaseous nature of H_2_S, poor thermal and aqueous stability, short half-life, and potential toxicity when present in excess (12). Thus, potential therapeutic applications for H_2_S donors cannot be translated into clinical therapeutic conditions without an efficient method to deliver this gasotransmitter at a safe, controlled rate. Drug delivery systems provide numerous benefits, including a wide range of administration techniques, increased efficacy and longer circulation times, and the potential for targeted delivery. Presently, few studies have focused on the formulation and delivery aspects of H_2_S and its donors. Instead, H_2_S usually is administered either by direct exposure to the animal through inhalation or via site-specific injections of donor solution (13, 14).

Alternative routes to oral drug administration such as the transdermal route have become more popular in recent years. There are many benefits associated with transdermal drug delivery systems (TDDS) over the inhalation route in that it allows more specific dosing, minimising the risk of toxicity whilst providing a more patient friendly option owing to the foul odour of H_2_S gas (15, 16). Furthermore, injections require a trained professional to administer the donor as well as being a painful route of drug administration. TDDS can also provide controlled drug release, steady-state plasma levels, improved bioavailability, and are patient friendly (17, 18). TDDS may include chemical permeation enhancers such as propylene glycol, oleic acid or surfactants which reversibly decrease skin barrier function by disrupting intercellular stratum corneum lipids (19). Topically applied nanoparticle mediated delivery systems including liposomes, ethosomes and deformable liposomes offer benefits including less permanent disruption to the stratum corneum as well a controlled release of drug (20–22). The inclusion of surfactants such as Tween 20 within the bilayer of deformable liposomes destabilises the vesicle bilayer by reducing the amount of membrane elastic energy required for the liposome to deform allowing the liposome to become more elastic thus increasing the flux across the skin (23–25). By being able to change shape and volume at minimal energetic cost, these structures may even penetrate across hydrophilic pathways of intact skin (26, 27).

The therapeutic use of H_2_S provides a unique challenge for chemists to develop safe H_2_S releasing compounds or formulation systems to give a safe and sustained release of H_2_S. In this study, we sought to investigate the feasibility of transdermal delivery of ADT-OH in an aqueous gel using penetration enhancer, propylene glycol (PG), or liposomes, and explored the ability of permeated ADT-OH to promote angiogenesis and its potential effects in preserving the mitochondrial bioenergetics in endothelial cells.

## Materials and Methods

### Materials

Soy phosphatidylcholine (PC) was obtained from Avanti Polar Lipids. Hydroxypropyl methylcellulose (HPMC) (catalogue #09963), polyethylene glycol (grade ≥ 99.5%, catalogue #W294004), cholesterol (grade ≥ 99%, catalogue #C8667) and Tween 20 (grade ≥ 95%, catalogue #P1379) were obtained from Sigma-Aldrich (Dorset, England). ADT-OH was obtained from Cayman Chemical Company (catalogue #17102). All other reagents including trifluoroacetic acid, ethanol and acetonitrile were obtained from Fisher Scientific. Ultrapure water was obtained from a Milli-Q purification system (Millipore, Billerica, MA, US). Polycarbonate filter, pore size 400 nm, 200 nm, 100 nm, and 50 nm, were obtained from Sigma-Aldrich.

### Murine Skin Tissue

Skin excises were obtained from sacrificed female mice (20–25 g). The hair was removed from the dorsal portion using an animal hair clipper. After harvesting the full thickness skin, the fat adhering on the dermis side was removed using a scalpel and isopropyl alcohol. All animal experiments were carried out using procedures approved by the Aston University Ethical Review Committee in compliance with the UK Home Office Licence Number 3003453 in accordance with the ‘Guidance on the operation of Animals’ under the United Kingdom Animals (Scientific Procedures) Act 1986.

### Determination of Solubility and Partition Coefficient

The supernatant of supersaturated solutions maintained at 37°C of ADT-OH in water and PBS was analysed through HPLC separation coupled with UV detection to determine solubility. The partition coefficient was determined for both water and PBS as a solvent. In both cases 1 part of water was mixed with 1 part of n-octanol in a glass vial (28). ADT-OH was added until saturation was apparent. Vials were shaken at 100 RPM for 24 h at 37°C and they were left to settle for another 24 h. The layers were then separated and analysed via HPLC separation coupled with UV detection.

### Deformable Liposome Preparation

Liposomes were prepared by the ethanol injection method established by Batzri and Korn, 1973 (29). Briefly egg PC and cholesterol (16:8 μM) and 100 μg ADT-OH were dissolved in ethanol. Deformable liposomes were prepared with the addition of 10 w/w % Tween 20 of the lipids at the lipid mixing stage. The resulting organic phase was injected by means of a syringe pump in 1 mL of distilled water under magnetic stirring at a temperature above the transition temperature of the lipids (25°C). The liposome suspension was continually stirred for 5 min at room temperature. The resulting mixture was extruded sequentially 8 times through a 400-nm, 200-nm, and 100-nm diameter polycarbonate membranes, using an Avanti Mini Extruder to produce unilamellar vesicles. The ethanol and unentrapped drug was removed by dialysis against distilled water over 24 h using Slide-A-Lyzer dialysis cassettes. The mean liposome size, polydispersity index (measurement of the level of homogeneity of particle sizes) particle charge and deformability of liposomes was assessed used established protocols in our laboratory as described before (30).

### Differential Scanning Calorimetry of ADT-OH and ADT-OH Gel Formulations

To assess thermal characteristics of materials including melting temperatures, phase transitions and heat capacity changes of ADT-OH and ADT-OH gel formulations were analysed using a TA Instruments Q200 Thermal Analysis Differential scanning calorimetry (DSC). 2 mg of ADT-OH or formulation was weighed into T-Zero aluminium pans and then hermetically sealed. All experimental runs commenced at an initial temperature of 0°C, purged under nitrogen gas, with a scan rate of 10°C/min to 300°C.

### HPLC Methodology

Detection of ADT-OH was assessed using a reverse phase HPLC method adapted from (14). A Shimadzu LC-2030C Plus RoHS - Prominence-I separation module HPLC with UV detection was utilised at an operating wavelength of 435 nm. A Phenomenex HyperClone™ column (5 μm C18 4.6 × 150 mm column) was used with a 10 μL sample injected at 27°C. The mobile phase comprised of a 64:36 ratio of acetonitrile to 0.05% TFA in water at a flow rate of 1.25 mL/min. Stock solutions and standard solutions of ADT-OH were prepared in PBS ranging from 250 to 10 μ/mL. A final calibration curve with an R^2^ of 0.98 and a linear equation of y = 3 × 10^7^ was obtained.

### Determination of Liposome Entrapment Efficiency

The entrapment efficiency of ADT-OH in liposomes was determined following comparison of drug concentration in liposome samples pre and post dialysis. Liposome bilayer was disrupted following the addition 9 parts of acetonitrile to 1 part of liposome formulation. The mixture was centrifuged at 16000RCF from which the concentration in the supernatant samples was assayed. HPLC-UV analysis was used to determine the encapsulation efficiency of ADT-OH in liposomal formulations (Eq. 1):

1 $$ E=\frac{D_t-{D}_s}{D_t}\times 100\% $$

where *E* is the encapsulation efficiency (%), *D*_*t*_ is the total drug content (mg), and *D*_*s*_ is drug content in supernatant (mg).

### ADT-OH Loaded Aqueous Gel Formulation

HPMC aqueous gels were prepared at 5% w/v in distilled and deionised water and mixed overnight using a mechanical mixer (Polytron PT 3100 D) at a speed of 3000 rpm. Gels with an ADT-OH loading of 0.025% w/w were manufactured with either 0, 2 or 10% PG or liposomes loaded with an equivalent quantity of ADT-OH. Final gel preparations all contained 15% v/v ethanol.

### pH Determination

Samples of each gel formulation were diluted 10-fold with distilled and deionised water prior to pH analysis (Sartorius Professional Meter PP-20).

### Gel Release Study on Excised Murine Skin

Permeation studies were carried out in vitro with Franz diffusion cells (PermeGear, Hellertown, PA, USA) for the assessment of PG or the liposomal gel’s ability to improve the flux of ADT-OH across skin. The system was maintained at 35°C by means of a shaking incubator set at 20 RPM to prevent any diffusion layer effects during the study (LSE, 49 L, Corning). The cells orifice diameter was 11.28 mm, the effective diffusion area was 1.00 cm^2^ and the receptor volume was 8 mL. The receptor was filled with PBS (pH 7.4). Murine skin (epidermis layer of average thickness 0.34 ± 0.02 mm) samples were cut, washed, and used immediately following excision. Skin samples were carefully mounted on the receptor chamber of a vertical Franz Diffusion cell. The receiver compartment was carefully filled with PBS:ethanol (95:5) to ensure no air bubbles next to the skin. After the assembled Franz cell was equilibrated for at least 30 min, 0.2 mL of each gel formulation was applied to the skin. Over the course of the study, the diffusion systems were shaken at 20 RPM to prevent any diffusion layer effects. At appropriate time intervals, 200 μl of the receptor medium was withdrawn, and the same volume of fresh buffer solution was replaced to the receptor chamber. The concentration of the samples was assayed with HPLC-UV as previously described.

### Effect of Permeation Enhancer Loading and Delivery Vehicle on ADT-OH Skin Deposition

Following the release study, skin sample was removed from the Franz cell and carefully washed 3 times in PBS. Samples were cut and weighed (≤30 mg) and immersed in acetonitrile containing ceramic beads. Tissue was homogenised using automatic homogeniser (automatic VelociRuptor V2 Microtube Homogeniser) in a room temperature. Samples were then removed and centrifuged at 16000 RCF from which the concentration in the supernatant samples was assayed with UV–Vis Spectrophotometer and HPLC-UV as previously described. The In-vitro drug release kinetics were assessed as described previously using Microsoft Excel®. Zero order, first order and Higuchi release profiles were applied to release from each formulation following which regression analysis techniques were employed to determine the probable drug-release(30). The release kinetic model displaying the highest r^2^ metric (≥ 0.95) was determined to be the mechanism, by which release occurred.

### Calculation of Flux and Permeability Coefficients

The lag time for the gel formulation can be calculated from the gel release study by extrapolating the tangent of the curve from the first 2 h to the X-axis; this line intersects with the time axis at some point where the amount of drug is zero; this time is called lag time (t_lag_). The steady-state flux (D_s_) (between 0.5 and 2 h) and permeability coefficients (K_p_) were obtained as shown using ${D}_s=\frac{h^2}{6{t}_{lag}}$ and ${K}_p=\frac{D_s{K}_{o/ pbs}}{h}$ where h is the epidermis thickness.

### HUVEC Cell Culture

Primary Human Umbilical Vein Endothelial cells (HUVEC, PromoCell, Cat. # C-12203) were cultured in full growth media (EGM-2) (PromoCell, Cat. # C-22211) supplemented with Fetal Calf Serum 0.02 mg/mL, Epidermal Growth Factor 5 ng/ml, Basic Fibroblast Growth Factor 10 ng/ml, Insulin-like Growth Factor 20 ng/mL, Vascular Endothelial Growth Factor 0.5 ng/mL, Ascorbic Acid 1 μg/mL, Heparin 22.5 μg/mL, Hydrocortisone 0.2 μg/mL (supplement kit, Promocell, Cat. # C-39211) and 5 mL of 1X Penicilin/Streptomycin (Lonza, Cat. # LZDE17-602E). Culture medium was changed every 48 h and cells were incubated at 37°C in a 5% CO_2_ humidified atmosphere incubator. Cells were sub-cultured at 70–80% confluency and used for experiments up to passage 5. Before each treatment cells were starved using 5% FBS (Gibco, Cat. # 11550356) M199 starvation media (Lonza, Cat. # LZBE12-119F) supplemented with antibiotics for 2 h. To ensure the quiescent state of the cells and consistent results, treatments were diluted in the starvation media unless otherwise stated.

### H_2_S Release from ADT-OH Permeated Across Murine Skin

The release of H_2_S from ADT-OH permeated across murine skin into cell culture media was measured over 24 h to observe the ability of the compound to release of the active the gasotransmitter moiety.

H_2_S production was then determined using a H_2_S-specific microsensor (ISO-H_2_S-100; World Precision Instruments, Sarasota, FL, USA) connected to a single channel free radical analyser (TBR 1025; World Precision Instruments). The sensor was set to the 100-nA range. The H_2_S calibration curve was created by preparing serial dilutions of freshly dissolved Na_2_S and by measuring the electrochemical response. The H_2_S generation is reported as hourly change in absorbance with respective H_2_S concentrations (31). Furthermore, as free H_2_S is strong reducing agent and reacts with the tetrazolium dye 3- (4,5-dimethyl-2-thiazolyl)-2,5-diphenyl-2H-tetrazolium bromide (MTT, Sigma) and forms purple colour formazan (32). Thus, H_2_S production was confirmed with this assay (Supplementary Methods).

### Mitochondrial Oxygen Consumption

Oxygen consumption rates (OCR) were evaluated using an XF24 Seahorse Extracellular Flux Analyser (Agilent-Seahorse). HUVEC cells were plated in V7 XF24 Seahorse cell culture plates at a density of 5 × 10^4^cells/well in 100 μL of standard EGM-2 growth media and let to attach overnight. Cells were then washed in non-buffered media (containing 10 mM glucose, 1 mM pyruvate and 2 mM glutamine), to allow temperature and pH equilibrium. OCR were measured to establish a baseline. Next, media collected following murine skin permeation studies after 24 h containing ADT-OH as well as a control sample with no ADT-OH was injected at available ports. Following which four measurements of OCR were taken during a period of 30 min. Next, a mixture of rotenone and antimycin A (R/AA) (0.5 μM) was injected to allow the inhibition of the mitochondrial electron transport chain (by inhibiting complexes I and III, respectively). The fourth OCR measurement after ADT-OH injection was used to calculate the relative OCR in response to acute ADT-OH. The non-mitochondrial respiration was measured as the lowest OCR measured after R/AA injection. The basal respiration was calculated by subtracting the ADT-OH OCR by the non-mitochondrial respiration.

### Tube Formation Study

HUVEC were seeded on 6-well plates and formation of capillary-like structures was examined on growth factor-reduced Matrigel in 24-well plates as described previously (33). Cells were labelled with Calcein AM dye (Thermo Fisher) and tube formation was quantified by measuring the total tube length in five random ×200 power fields per well using a Nikon phase-contrast inverted microscope with Image ProPlus image analysis software (Media Cybernetics, Silver Spring, USA). Mean total tube length was calculated using with ImageJ Angiogenesis Analyzer from three independent experiments performed in duplicate.

### Statistical Analysis

Unless otherwise stated, all results are presented as mean ± standard deviation (SD). Replicates of at least 3 were used for all studies. T test or one-way ANOVA was used to determine any statistically significant difference between means tested (*p* ≤ 0.05). A post-hoc Tukey’s multiple comparisons test was then applied to assess differences between groups. All the calculations were carried out using Graphpad 8 (GraphPad Inc., La Jolla, CA).

## Results

### Development and Evaluation of ADT-OH and Gel Formulations

Currently, the therapeutic potential of H_2_S donors is limited by their insolubility and rapid rate of release. HPMC gels for transdermal application loaded with ADT-OH were formulated to provide a novel solution. The physicochemical properties of ADT-OH were assessed first to guide formulation development. The apparent partition coefficients for ADT-OH in water and PBS pH 7.4 were determined to be 0.25 ± 0.04 mg/ml and 0.06 ± 0.01 mg/ml respectively (Table I). Other physiochemical parameters such as log P were also determined. A negative log P value indicates a higher affinity for the aqueous phase whereas a positive value denotes a higher concentration in the lipid phase. ADT-OH is a hydrophobic drug with low solubility in water and PBS solution of pH 7.4 (Table I).

**Table I:** Experimentally Determined Physicochemical Properties Determined ADT-OH is a Lipophilic Compound

Media	Parameter	Value
Water (37°C)	Solubility	0.25 mg/mL (±0.04)
	K_o/w_	617.77 (±36.65)
	LogP	2.78 (±0.02)
PBS (37°C)	Solubility	0.06 mg/mL (±0.01)
	K_o/w_	444.34 (±47.97)
	LogP	2.64 (±0.05)

Results are representative or expressed as mean ± standard deviation. *n* = 3 independent batches

Gels were formulated with increasing loading of the permeation enhancer propylene glycol (PG) (0–10% v/v) or liposomes loaded with ADT-OH. The final loading of ADT-OH remained constant at 0.025% w/v. Liposome physicochemical characteristics, such as size, polydispersity, zeta potential, entrapment efficiency, and deformability are important for drug release, permeation, and efficacy. These characteristics and the impact of the inclusion of Tween 20 within the deformable liposomal formulation (DL) on liposome characteristics were determined. In the presence of surfactant in the bilayer of liposomes did not significantly affect the liposome diameter, polydispersity or charge as defined by zeta potential (Table II). However, the entrapment efficiency of ADT-OH within the liposome significantly increased from 88.56 ± 10.06 to 94.89 ± 3.84% for conventional liposomes and those formulated with Tween 20 respectively (*p* < 0.05). The degree of liposome deformability determined by comparison of size pre and post extrusion through a 50 nm pore polycarbonate filter was defined as deformability index (DI). The DI assesses liposome ability to regain size after having been forced through a pore size smaller than their original diameter. The greater the degree of deformation the less elastic the liposomes are as they were unable to regain their previous larger size. The DI following extrusion was significantly lower in deformable liposomes compared to conventional liposomes (19.73 ± 6.51 *vs* 39.89 ± 5.35) (*p* < 0.01). Only deformable liposomes were taken forward to produce gel formulations.

**Table II:** Inclusion of Tween 20 in the Liposome Formulation Improves Liposomal Characteristics Compared with Conventional Liposomes

	Size (nm)	Polydispersity	Zeta potential	Entrapment efficiency	Deformability index
Liposomes	139.75 (±5.26)	0.20 (±0.02)	−17.03 (±5.95)	79.91 (±7.13)	39.89 (±5.35)
DL	127.82 (±7.05)	0.19 (±0.02)	−22.20 (±1.00)	94.89 (±3.84)	19.73 (±6.51)

Liposome size distribution, polydispersity, zeta potential, entrapment efficiency and deformability index for liposomes and deformable liposomes (DL) formulations

Liposomes were prepared by the ethanol extrusion method with ADT-OH added during the lipid mixing stage. Data represents mean ± standard deviation. *n* = 3 independent batches

**Fig. 1:**
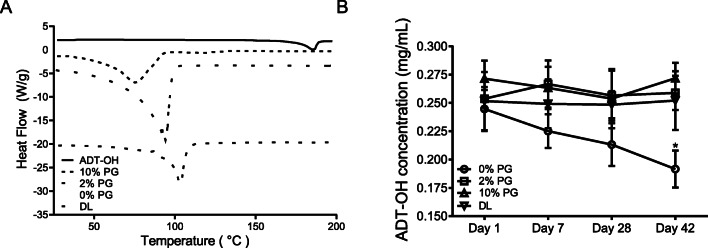
Inclusion of up to 10% v/v PG and/or inclusion of DL in gel formulation improves solubility and stability of ADT-OH (0.025% w/w) within the formulation. (**A**) Differential scanning calorimetry analysis scans of ADT-OH alone and when combined in gel formulations. All experimental runs started at an initial temperature of 0°C, purged under nitrogen gas, with a scan rate of 10°C/min to 300°C. (**B**) Stability of drug in gel formulation over 42 days as determined by ADT-OH concentration. Results are representative or expressed as mean ± standard deviation and analysed by One-way ANOVA. *n* = 3 independent batches. *p < 0.05.

Drugs with melting point of <200°C are better poised to cross the subcutaneous layer (21). The melting point (T_m_) of ADT-OH was at 185°C (Fig. 1A). Differences between reported values and those observed here may be due to differences in ADT-OH sample purity. Furthermore, the T_m_ of ADT-OH disappeared following all formulation designs (the visible peaks between 80 and 105°C are all due to water evaporation) indicating complete solubilisation. The stability of the ADT-OH in the gel formulation was assessed by measuring drug concentration over 42 days with HPLC-UV detection (Fig. 1B). Drug concentration was maintained in all formulation except the gel formulated with no PG where a decrease was observed over this time period (*p* < 0.05). The physiological pH of the stratum corneum is 4.1–5.8 (33) thus an ideal transdermal formulation should fall within this range to avoid skin irritation. The pH of all formulations were within this range (Supplementary Table 1).

### Influence of Permeation Enhancers on the Flux of ADT-OH Through Excised Mouse Skin

**Fig. 2:**
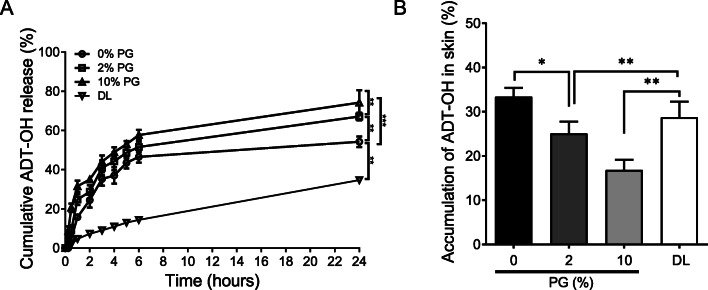
Increasing PG loading to 10% v/v in gels loaded with 0.025% w/wADT-OH increased ADT-OH flux across *ex-vivo* murine skin samples and reduced skin retention. (**A**) Cumulative percentage ADT-OH release profiles up to 24 h. (**B**) ADT-OH accumulation in *ex-vivo* murine skin samples following 24-h application to a 1 cm^2^ sample of excised skin. Release across skin was observed using a Franz cell system quantified by HPLC-UV analysis. Results are representative or expressed as mean ± standard deviation and analysed by One-way ANOVA. *n* = 3 independent batches. ***p < 0.001, **p < 0.01.

Having established the transdermal formulations, the permeation of ADT-OH through *ex vivo* samples of murine skin were assessed for each gel formulation. 24 h after the applications of ADT-OH gels formulated with 0, 2, 10% v/v of PG, and deformable liposome on the skin, the percentages of permeated ADT-OH were 54.2 ± 2.7%, 67.1 ± 1.9%, 74.2 ± 6.4% and 34.65 ± 1.25% respectively (Fig. 2A). A significant difference across all formulations (*p* < 0.001) was observed across the time points. Specifically, the 24-h time point, PG significantly increased the cumulative ADT-OH release across the skin in a concentration dependent manner (*p* < 0.05 for 0 and 2% v/v PG, *p* < 0.001 for 0 and 10% v/v PG) (Fig. 2A). Furthermore, compared to PG formulated gels, liposome formulation resulted in a slower rate of drug permeation through the skin (Fig. 2A and Table III). Following 24-h application of each formulation, accumulation of ADT-OH in *ex vivo* skin samples were measured. In contrast, a reversed pattern of the ADT-OH levels were observed (Fig. 2B). Specifically, results showed that the higher concentration of PG in the formulations, the lower levels of ADT-OH were detected in the skin. No significant differences between 0% PG gel and deformable liposomal gel were observed.

**Table III:** Release of ADT-OH from Gels Proceeds by Diffusion and Obeys the Higuchi Law

Kinetic model	Parameter	HPMC gel formulated with PG			DL HPMC gel
		0% v/v	2% v/v	10% v/v	
0	k_0_ (mg.min-1)	3.81 (±0.23)	3.95 (±0.09)	4.03 (±0.24)	1.61 (±0.04)
	R^2^	−0.31 (±0.15)	−0.63 (±0.23)	−0.83 (±0.34)	0.83 (±0.05)
	AIC	79.33 (±1.60)	80.90 (±0.81)	80.31 (±0.33)	68.28 (±0.64)
1st	k_1_ (min^−1^)	0.14 (±0.02)	0.16 (±0.01)	0.15 (±0.01)	0.02 (±0.001)
	R^2^	0.72 (±0.08)	0.63 (±0.09)	0.55 (±0.17)	0.90 (±0.03)
	AIC	65.27 (±1.22)	66.67 (±1.57)	67.17 (±2.51)	63.39 (±1.74)
Higuchi	k_H_	17.46 (±1.15)	17.71 (±0.35)	19.52 (±0.43)	6.31 (±0.09)
	R^2^	**0.72 (±0.03)**	**0.69 (±0.04)**	**0.68 (±0.11)**	**0.94 (±0.02)**
	AIC	65.42 (±1.97)	65.15 (±0.70)	65.76 (±2.94)	45.14 (±5.81)

ADT-OH release from the transdermal gel was modelled using various mathematical laws including zero order kinetics, first order kinetics and Higuchi. As PG loading increased, an increase in the rate constant was observed whereas DL caused a decrease. R^2^, coefficient of determination; AIC, Akaike Information Criterion; F is the fraction of drug released at time t; k0 is the zero-order release constant; k1 is the first-order release constant; k_H_ is the Higuchi release constant. Bold text indicates the highest R^2^ value. Results are presented as the mean ± standard deviation (*n* = 3)

Using the determination coefficient (R^2^) and Akaike Information Criterion (AIC), the model that best described ADT-OH release from the gel formulations was the Higuchi model (highest R^2^ and lowest AIC) (Table IV). Using this model, the Higuchi dissolution constant, K_H_ was significantly increased in 10% PG formulated gel compared to 0% and 2% PG gels (0% v.s 2% v.s 10% PG: 17.46 ± 1.15, 17.71 ± 0.35 and 19.52 ± 0.43 respectively *p* < 0.05). Interestingly, K_H_ value was significantly reduced in liposomal gels (6.31 ± 0.09 *p* < 0.0001) compared to all the other formulations.

**Table IV:** Experimentally Determined Coefficients Observed Increasing PG Loading to 10% Causes an Increase in ADT-OH Flux and Permeability Whereas DL Causes a Reduction in These Parameters

Gel formulation	0% v/v PG (a)	2% v/v PG (b)	10% v/v PG (c)	DL (d)
T_lag_ (hours)	0.102 (±0.007)^Ø#^	0.076 (±0.006)^^‡^	0.067 (±0.006)^¥^	0.183 (±0.015)
Skin diffusion coefficient D_s_ (mg/mm^2^/h)	0.20 (±0.01)^*Ø #^	0.26 (±0.02)^‡^	0.30 (±0.03)^¥^	0.11(±0.01)
Permeability coefficient K_p_(mm/h)	252.97 (±18.61)^*Ø #^	340.06(±26.82)^‡^	386.59 (±36.58)^¥^	140.40(±11.43)

^*^Denotes a difference between group a and b. ^Ø^Denotes a difference between group a and c. ^#^Denotes a difference between group a and d. ^^^Denotes a difference between group b and c. ^‡^Denotes a difference between group b and d. ^¥^Denotes a difference between group c and d. Differences were deemed significant when *p* < 0.05

Experimentally determined coefficients used to quantify and compare drug permeation across the skin from the various formulations are shown in Table IV. The t_lag_ decreased with increasing PG loading and is highest in the liposomal gel formulation. The D_s_ and K_p_ increased with increasing PG loading and was lowest in the liposomal gel formulation. The flux (D_s_) increased by a factor of 1.5 between 0 and 10% v/v PG (*p* < 0.001). However, the flux of the liposomal formulation decreased significantly by a half compared with the gel with no penetration enhancer (*p* < 0.01). Further, the permeability coefficient was significantly lower in the liposomal gel compared with all other formulations (*p* < 0.01 compared with PG 0% v/v, and *p* < 0.0001 with PG 2 and 10% v/v). In line with the parameters established, the K_p_ also significantly increased as the loading of PG increased (*p* < 0.05 between 0 and 2% v/v PG loading, and *p* < 0.001 between 2 and 10% v/v loading).

### Effects of Permeated ADT-OH on Endothelial Cell Functions

**Fig. 3:**
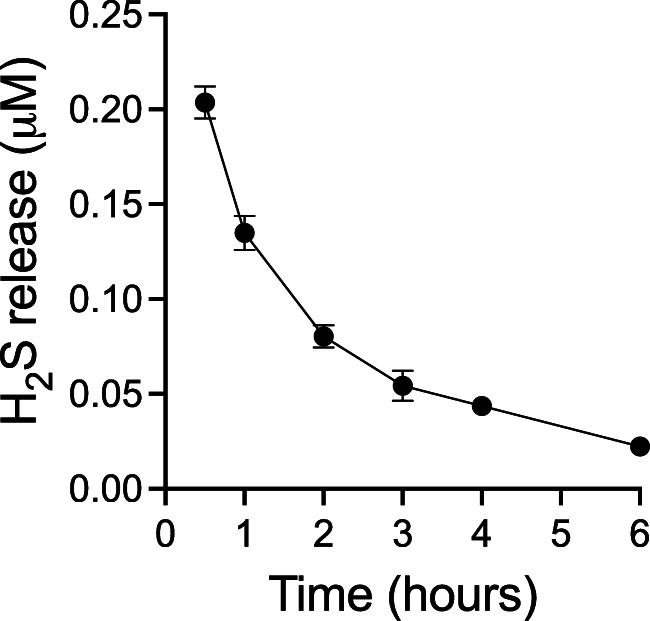
H_2_S is rapidly released from ADT-OH on HUVEC cells. Hourly H_2_S release values detected using a H_2_S probe are plotted with curve-fitting results to highlight the donor compound decomposition on HUVEC cells.

To confirm that the permeated ADT-OH retained the ability to release H_2_S, HUVEC cells were incubated with conditioned media containing ADT-OH permeated across murine skin from gels formulated with 10% PG, and the levels of H_2_S were measured over 6 h. Maximum release of H_2_S was detected within 30 min of exposure to HUVEC (Fig. 3 and Supplementary Fig. 1). The levels of H_2_S in the media decreased rapidly. By 6 h, H_2_S levels were reduced to a minimum level.

**Fig. 4:**
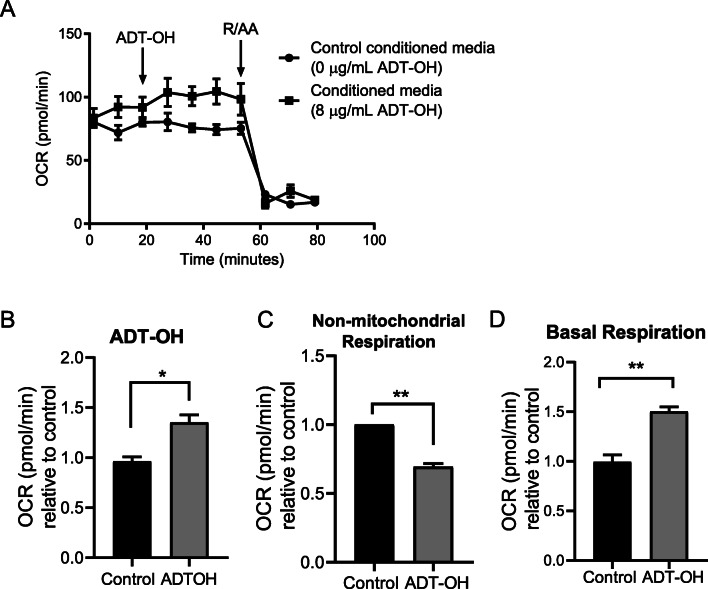
Functional properties of permeated ADT-OH formulated with 10% v/v PG enhances mitochondrial respiration. (**A**) Oxygen consumption rates (OCR) expressed by time in HUVEC cells exposed to ADT-OH followed by inhibition of mitochondrial complexes I/III using a mixture of rotenone and antimycin A (R/AA). (**B**) OCR relative to control measured after 30 min of exposure to ADT-OH. (**C**) OCR relative to control measured after the inhibition of complexes I/III. (**D**) Basal respiration relative to control (measured as the difference between ADT-OH–Non-mitochondrial respiration). Results are representative or expressed as mean ± standard deviation and analysed by T test. *n* = 3 independent batches. **p < 0.01, *p < 0.05.

To investigate the bioactivities of transdermal preparation of ADT-OH, we then assessed the effect of permeated ADT-OH across murine skin on the mitochondrial respiration in endothelial cells using seahorse analysis. HUVEC cells were exposed to media collected following murine skin permeation studies after 24 h containing ADT-OH or vehicle and real-time OCR was measured at 10 min intervals over 80 min (Fig. 4A). Acute ADT-OH exposure was observed to significantly improved the OCR thus enhanced the mitochondrial respiration by 0.4-fold (*p* < 0.0001) (Fig. 4B), whilst it dramatically decreased the non-mitochondrial respiration in HUVEC cells (Fig. 4C). In addition, the basal respiration (Fig. 4D) increased by 0.5-fold in the cells exposed to media containing ADT-OH suggesting that the transdermal preparation of ADT-OH is able to exert protective effects on the mitochondrial cellular bioenergetics.

**Fig. 5:**
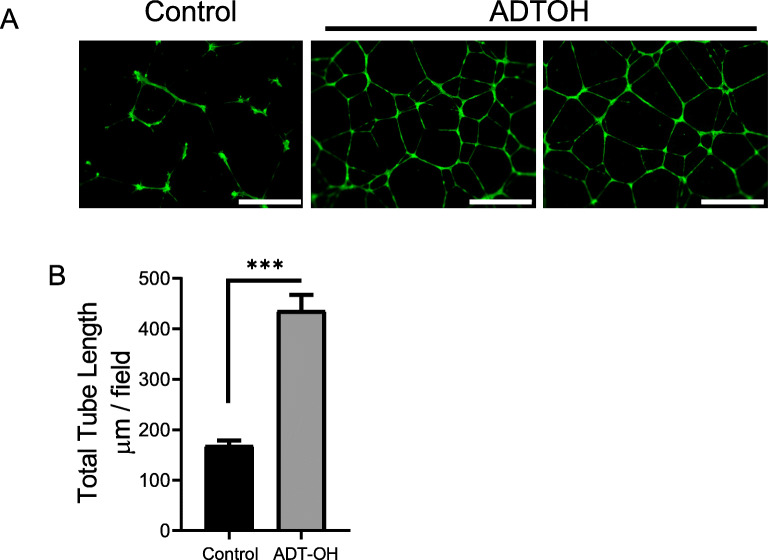
Permeated ADT-OH increased total tube length in HUVEC cells. Media from the permeation studies from gels formulated with 10% v/v PG was selected for the tube formation assays to test functionality of ADT-OH following permeation across the excised murine skin samples. (**A**) Fluorescent imaging of tube formation assay. (**B**) total tube length comparing control HUVEC with ADT-OH treated HUVEC. Results are representative or expressed as mean ± standard deviation and analysed by T test. *n* = 3 independent batches. ***p < 0.001. Scale bar: 100 μM.

Furthermore, incubation with conditioned media containing ADT-OH in HUVEC cells led to an increased level of tube formation compared to vehicle controls (Fig. 5). Tube length increased from 169 ± 29 μM to 436 ± 94 in HUVEC cells treated with media containing vehicle or ADT-OH respectively (*p* < 0.05).

## Discussion

Hydrogen sulphide (H_2_S) has been increasingly recognised as a key player in many physiological and pathophysiological processes highlighting its therapeutic potential in the treatment of a range of diseases. Over the last decades, many new classes of H_2_S donors have been developed, however, very few have reached the clinic. The major challenge concerns the delivery of H_2_S donors at a controlled rate to ensure a maximum H_2_S bioavailability and a minimal potential toxic effect of the by-products. In this study we, for the first time, developed a transdermal delivery system for a H_2_S donor; ADT-OH. We provided evidence that ADT-OH can gradually flux across ex-vivo murine skin and that the permeated ADT-OH is able to enhance mitochondrial respiration and tube formation in HUVECs. This delivery system of H_2_S donor has the potential to avoid repeated dosing, drug instability concerns or first pass metabolism with oral delivery systems, and ultimately reduce adverse effects.

Ethanol and PG are commonly used solvents and/orco-solvents for transdermal formulations (34). Ethanol has preservative properties however can also disrupt the cutaneous barrier function causing lipid fluidization and extraction therefore limiting resistance to drug moving through the skin (35). PG is commonly used in skin preparations either as a co-solvent for poorly soluble materials and/or to enhance drug permeation through the skin from topical preparations. Mechanisms of action of PG in enhancing drug permeation include a carrier-solvent effect (36), α-keratin structure solvation (37), penetration via dehydration (38) and an increase in solubility and disorder of intercellular lipids of stratum corneum (39). Indeed, HPMC carrier formulated with PG not only increased the solubility but also enhanced the stability of ADT-OH within the gel formulation. Further, increasing loading of PG within the formulation was able to increase the transdermal flux rate of ADT-OH in a concentration dependent manner. The release of ADT-OH from different formulations was in favour of the Higuchi diffusion model demonstrating the diffusion-controlled mechanism of ADT-OH release. Therefore, the amount of drug released was proportional to the square root of time for all four formulations and the rate of release is proportional to square root of drug solubility, exposed surface area, diffusion constant and inverse time (40). In addition, increasing level of PG in the formulation decreased drug deposition in the skin indicating that PG not only allowed ADT-OH to enter the skin, but was able to pull drug across the skin. Our results are in tandem with another study that showed a correlation between the amount of active, loperamine, permeated and amount of PG (15 and 40% v/v) dosed on the skin (41). Although drug penetration through murine skin may differ from that in human tissue, the latter is not readily available. Murine skin is a widely used alternative (42–45) with some compounds permeating in a similar manner, whereas others differ in drug release owing to human skin being less permeable (46). Further work is required to establish the drugs kinetic profile thought human skin. Nonetheless, permeated ADT-OH stimulated tube formation and enhanced the mitochondrial respiration in endothelial cells, suggesting that the bioavailability of ADT-OH and its protective cellular effects can be achieved using transdermal formulations.

Nanoparticle delivery systems are a relatively new technology, but it has been increasingly recognised as a valuable transdermal drug delivery carrier. Liposomes are well-established nanocarriers that have been proven to be effective in topical and transdermal drug delivery (47). Liposome physicochemical characteristics, such as size, charge, composition, and the stability play important roles in liposome mediated drug delivery. For example, a liposome preparation homogenous in size, as observed in this study, is important for drug release kinetics and the degree of tissue distribution *in vivo*(48, 49). Further, a neutral liposomal surface charge can reduce skin irritation (50) however, a slight charge, as demonstrated in this study, is useful to avoid particle flocculation due to electrostatic repulsion between liposomes during storage (51). Additional components such as Tweens, the edge-activators, in the liposome is able to diminish the energy required for particle deformation and accommodate particle shape changes of the liposomes under stress (52). Therefore, these deformable liposomes can access the viable epidermis by overcoming the physical constraints imposed by the stratum corneum (53). Interestingly, the DL gel formulation of ADT-OH gave the slowest rate of permeation as well as the lowest concentration of drug in the receiver compartment following 24 h. In addition, the DL formulation showed less deformation compared with the standard liposome following extrusion implying their bilayer had more elastic potential energy. Despite the potential for excess energy in liposomes formulated with Tween 20, these liposomes cannot fully regain their pre-extrusion size due to energy will always be loss as friction (54). Increasing the loading of Tween 20 or the use of other surfactants may be able to increase drug permeation across the skin. Nevertheless, this formulation may provide a therapeutic option for a low dose, controlled release formulation. The therapeutic efficacy of such formulations needs to be explored in future work.

H_2_S acts as a physiological mediator with a wide range of functions, such as angiogenesis and anti-inflammation(3). At low concentrations, H_2_S acts as an electron donor to stimulate the mitochondrial respiration (55). It has been demonstrated that oxidation and elimination of H_2_S at mitochondrial matrix can promote ATP synthesis and therefore enhancing the consumption of oxygen (56). Once H_2_S is released, it is rapidly oxidized and excreted as S_2_O_3_^−^ and SO_4_^2−^(57). Therefore, maintaining the chemical stability is important in developing transdermal delivery systems for H_2_S donors. We found that transdermal gels formulated with PG or DL greatly improved ADT-OH stability. Further, permeated ADT-OH can not only release H_2_S in HUVEC cells, but it can also enhance the mitochondrial activity and tube formation in these endothelial cells. Taken together, these findings demonstrated, for the first time that the transdermal delivery of ADT-OH allows a sustained rate of delivery with retained drug functionality.

## Conclusion

Transdermal delivery of ADT-OH is a promising non-invasive therapeutic option in the treatment of various cardiovascular conditions with the potential to offer sustained H_2_S levels in the circulation thus reduce toxicity and dose frequency. Aqueous gels formulated with increasing amounts of propylene glycol (0–10% v/v) were able to maintain the stability of ADT-OH over 6 weeks and increased ADT-OH permeation across excised murine skin whilst liposomal gel formulations were able to provide a slow-release option. Furthermore, permeated ADT-OH stimulated tube formation, increased oxygen consumption and enhanced mitochondrial function in HUVEC cells demonstrating potential for promoting endothelial cell functions. Therefore, the development of systemic delivery of H_2_S donors through the application to the skin appears to be a desirable and feasible alternative to oral or intravenous delivery.

## Supplementary Information

ESM 1 (PDF 71 kb)
