# Supplementary material for: Transdermal Delivery of a Hydrogen Sulphide Donor, ADT-OH Using Aqueous Gel Formulations for the Treatment of Impaired Vascular Function: an Ex Vivo Study
Source: Pharm Res. 2022 Jan 27;39(2):341–52. doi: 10.1007/s11095-021-03164-z (PMC8881439; doi:10.1007/s11095-021-03164-z)
Supplement: Supplementary file 1 — (PDF 71 kb) [file 11095_2021_3164_MOESM1_ESM.pdf]

## Supplementary Information

Methods

### $H_2S$ release from ADT-OH permeated across murine skin

Free $H_2S$ is strong reducing agent and reacts with the tetrazolium dye 3- (4,5-dimethyl-2-thiazolyl)-2,5-diphenyl-2H-tetrazolium bromide (MTT, Sigma) and forms purple colour formazan (32). Media collected following murine skin permeation studies containing was diluted 1 in 2 with fresh cell culture media and added to HUVEC cells. Over the course of 6 hours, 100 µL of media was removed and was added to 50 µL MTT (5 mg/mL), allowed to incubate for 3 hours and changes in absorbance were recorded on a plate reader at 570 nm. The reaction was carried out in a humidified incubator at 37 °C with 5% $CO_2$ atmosphere to mirror the cell culture conditions and minimise evaporation. The $H_2S$ calibration curve was created by preparing serial dilutions of freshly dissolved $Na_2S$ and by measuring the reducing capacity. The $H_2S$ generation is reported as hourly change in absorbance with respective $H_2S$ concentrations.

Figure 1.

**$H_2S$ is rapidly released from ADT-OH on HUVEC cells.** Hourly $H_2S$ release values detected using an MTT assay are plotted with curve-fitting results to highlight the donor compound decomposition on HUVEC cells.

Table 1. pH of all formulated HPMC gels loaded with up to 10% v/v PG or liposomes to give a final loading of 0.025% ADT-OH observed values similar to that of human skin.

Supplementary Figure 1

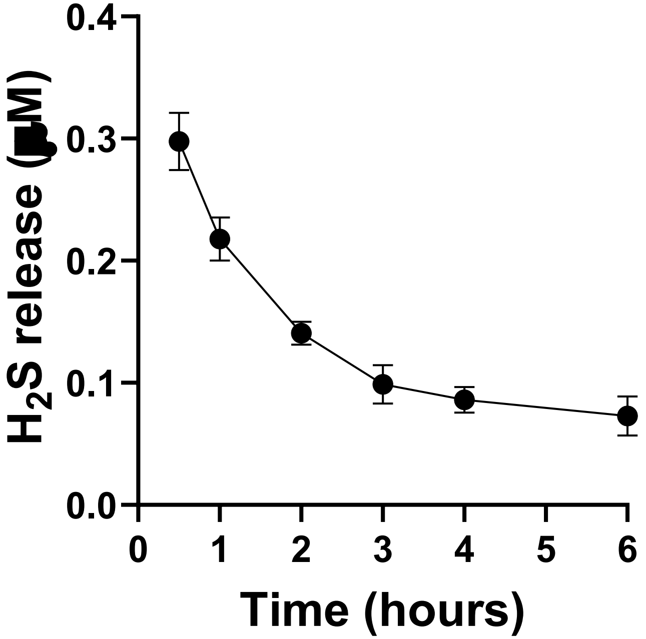

# Supplementary Table 1

| Formulation | pH |
| --- | --- |
| 0 % v/v PG | 5.5 ± 0.1 |
| 2 % v/v PG | 5.67 ± 0.06 |
| 10 % v/v PG | 5.77 ± 0.06 |
| Deformable liposomes | 5.8 ± 0.1 |
